# Diagnostic Accuracy of Magnetic Resonance Urography Compared With Computed Tomography Urography in Patients With Hematuria

**DOI:** 10.7759/cureus.114323

**Published:** 2026-08-11

**Authors:** Rajshree Ganjeer, Vijendra Ruprela, Baidya Nath Singh, Kashi Nath Sarkar, Amit Kumar, Prashant Gajanan Pote, Tushar Dani, Janganagari Anil, Roopshree Bode, Davidson Shandilya

**Affiliations:** 1 Department of Radiodiagnosis, Raipur Institute of Medical Sciences, Raipur, IND; 2 Department of General Surgery, Raipur Institute of Medical Sciences, Raipur, IND

**Keywords:** computed tomography urography, diagnostic accuracy, hematuria, magnetic resonance urography, radiology, urinary tract imaging, urolithiasis, urothelial carcinoma

## Abstract

Background

Hematuria is an important clinical finding that may reflect a wide spectrum of urinary tract abnormalities, ranging from infection and urolithiasis to renal masses and urothelial malignancy. CT urography (CTU) is widely used for the evaluation of hematuria, but magnetic resonance urography (MRU) offers a radiation-free alternative. This study aimed to evaluate the diagnostic performance of MRU compared with CTU in patients presenting with hematuria.

Methods

This prospective cross-sectional observational diagnostic accuracy study included 35 patients with hematuria who underwent both CTU and MRU as part of their diagnostic evaluation at a tertiary care teaching hospital over 18 months. Imaging findings were analyzed for lesion detection and etiological classification. The final diagnosis was established using a composite reference standard based on imaging findings, clinical assessment, laboratory data, and available operative, histopathological, or follow-up information. Sensitivity, specificity, positive predictive value, negative predictive value, diagnostic accuracy, and concordance between CTU and MRU were assessed.

Results

The study population included 21 males and 14 females, with a mean age of 45.09 ± 10.91 years. The most frequent etiologies were infection in nine patients, renal mass in eight patients, urothelial carcinoma in seven patients, and urolithiasis in six patients. CTU identified all 30 patients with urinary tract pathology and correctly classified all five patients without demonstrable pathology in this cohort. MRU identified 26 of 30 patients with urinary tract pathology and correctly classified all five patients without demonstrable pathology. MRU showed a sensitivity of 86.7%, specificity of 100.0%, positive predictive value of 100.0%, and negative predictive value of 55.6%. Agreement between CTU and MRU was substantial, with an observed agreement of 88.6% and a Cohen’s kappa coefficient of 0.65. Discordant cases were positive on CTU but negative on MRU, mainly reflecting reduced detection of selected urothelial lesions on MRU.

Conclusions

In this cohort, CTU showed the highest diagnostic performance for evaluating hematuria and provided comprehensive detection across the observed etiological spectrum. MRU demonstrated high specificity and substantial concordance with CTU, supporting its role as a useful radiation-free alternative in selected patients, particularly when radiation exposure or iodinated contrast administration is a concern. However, negative MRU findings should be interpreted with caution when clinical suspicion remains high. A patient-specific imaging approach may help balance diagnostic yield with safety considerations.

## Introduction

Hematuria is a common but clinically important presentation in urology, nephrology, and radiology. It may be visible to the patient as gross hematuria or detected only on urine microscopy as microscopic hematuria. Although some causes are benign and self-limiting, hematuria may also be the first manifestation of clinically significant urinary tract disease, including urolithiasis, infection, renal parenchymal disease, urothelial tumors, renal masses, trauma, or obstructive pathology [1,2]. A structured evaluation is therefore necessary, particularly in patients with persistent, recurrent, painless, or unexplained hematuria [1,2].

Imaging plays a central role in determining the site and cause of hematuria. Ultrasonography is often used as an initial investigation because it is widely available, inexpensive, and free of ionizing radiation. However, ultrasonography may be limited in the evaluation of small ureteric lesions, subtle urothelial abnormalities, and complex renal or collecting-system pathology [2]. Cross-sectional imaging is therefore frequently required when a more comprehensive evaluation of the kidneys, ureters, and urinary bladder is needed [3-6].

CT urography (CTU) is widely used for the radiological evaluation of hematuria because it provides rapid, high-resolution assessment of the renal parenchyma, collecting systems, ureters, and bladder [3,6,7]. Its multiphasic protocol enables the detection of calculi, renal masses, urothelial lesions, obstructive uropathy, and extraurinary findings that may influence management [3,7,8]. Prior CTU studies have also reported its diagnostic value in detecting urinary tract abnormalities, upper tract urothelial neoplasms, bladder cancer, transitional cell carcinoma, and urinary tract neoplasms in patients with hematuria [9-13]. Despite these advantages, CTU has important limitations. It involves ionizing radiation, often in multiple phases, and usually requires iodinated contrast administration. These factors are relevant in younger patients, patients requiring repeated imaging, pregnant patients, patients with contrast allergy, and patients with impaired renal function [5,6].

Magnetic resonance urography (MRU) has emerged as a radiation-free alternative for urinary tract evaluation [14-16]. Heavily T2-weighted static-fluid MRU can depict the collecting systems and ureters without contrast, while contrast-enhanced excretory MRU can provide anatomical and functional information regarding renal excretion and urothelial enhancement [14-17]. MRU also offers superior soft-tissue contrast, which can be useful in the assessment of renal masses, inflammatory lesions, periureteric disease, upper tract urothelial carcinoma, and obstructive uropathy [14-20]. However, MRU is not a complete replacement for CTU in all clinical situations. It is more time-consuming, less widely available in many centers, more susceptible to motion artifacts, and less sensitive for detecting small calculi than CTU [6,14,16,19,20].

The choice between CTU and MRU is therefore not simply a question of which modality is superior. Rather, the clinically relevant question is whether MRU can provide diagnostically reliable information in selected patients with hematuria while reducing radiation exposure and avoiding iodinated contrast when necessary [6,14,19,20]. Evidence comparing CTU and MRU in real-world hematuria evaluation remains limited, especially in resource-variable tertiary care settings where patient selection, modality availability, and clinical decision-making may differ from guideline-based ideal conditions [19,20]. Hence, the primary objective of this study was to evaluate the patient-level diagnostic performance of MRU in detecting clinically relevant urinary tract pathology in patients with hematuria, using CTU as the principal comparator within the same cohort. Secondary objectives were to compare lesion and etiological detection between CTU and MRU and to assess their concordance. By performing both examinations in the same patients, the study aimed to compare their relative diagnostic performance and explore the role of MRU as a radiation-free alternative or complementary modality in selected patients with hematuria.

## Materials and methods

Study design and setting

This was a prospective, cross-sectional, observational diagnostic accuracy study conducted in the Department of Radiodiagnosis at Raipur Institute of Medical Sciences, Raipur, Chhattisgarh, India. The study evaluated patients presenting with hematuria who underwent both CTU and MRU as part of their diagnostic workup. The purpose was to compare the diagnostic performance and concordance of MRU with CTU in identifying urinary tract abnormalities and the etiological causes of hematuria. The study was conducted over 18 months, from August 15, 2024, to February 14, 2026, after obtaining approval from the Institutional Ethics Committee. Eligible patients were enrolled consecutively after providing written informed consent and underwent imaging evaluation according to the institutional diagnostic protocol.

Study population

The study population comprised patients with hematuria referred for imaging evaluation from the departments of Surgery and Urology. Both outpatients and inpatients were considered eligible. Patients were included if they presented with hematuria, underwent both CTU and MRU during the diagnostic evaluation, had relevant clinical, laboratory, and imaging records available for analysis, and provided written informed consent for participation.

Pregnant patients, patients with severe contrast allergy, patients unable to undergo CTU or MRU because of contraindications such as claustrophobia or metallic implants, patients with acute renal failure or increased risk of contrast-related renal complications, patients with unstable medical conditions, patients with prior nephrectomy or major urological surgery, patients with incomplete records, and patients with purely medical non-urological causes of hematuria were excluded. The inclusion and exclusion criteria used for patient selection are summarized in Table 1.

**Table 1:** Patient selection criteria CTU: computed tomography urography; MRU: magnetic resonance urography

Inclusion criteria	Exclusion criteria
Patients presenting with gross or microscopic hematuria	Pregnant patients or patients in whom radiation exposure was clinically inappropriate
Patients referred for radiological evaluation of the urinary tract	Patients with severe iodinated contrast allergy, gadolinium-based contrast allergy, or high risk for contrast-related complications
Patients who underwent both computed tomography urography and magnetic resonance urography	Patients with contraindications to magnetic resonance imaging, including incompatible metallic implants, pacemakers, or severe claustrophobia
Availability of relevant clinical, laboratory, CTU, and MRU data	Patients with acute renal failure, unstable medical condition, or inability to complete the imaging protocol
Written informed consent obtained before inclusion in the study	Patients with prior nephrectomy, major urological surgery, incomplete records, or purely medical non-urological causes of hematuria

Sample size and sampling method

A total of 35 eligible patients were included using consecutive sampling during the predefined 18-month study period. All patients who fulfilled the inclusion and exclusion criteria and completed both CTU and MRU were enrolled. No formal prespecified sample-size calculation was performed. Therefore, the study should be considered an exploratory prospective paired diagnostic comparison, and the precision of the diagnostic accuracy estimates is limited by the relatively small sample size.

Imaging protocol

All included patients underwent CTU according to the standard departmental protocol, based on established principles of multiphasic CT urography described in prior literature [4,6,7]. CTU was performed to evaluate the kidneys, collecting systems, ureters, and urinary bladder. The protocol included non-contrast imaging followed by contrast-enhanced phases as clinically appropriate, including nephrographic and excretory phase imaging. CTU findings were assessed for calculi, renal masses, urothelial lesions, obstructive changes, infective or inflammatory abnormalities, and other urinary tract pathologies.

The same patients subsequently underwent MRU using a predefined MRI protocol, based on established static-fluid and excretory MRU techniques described in prior literature [4,5]. MRU included heavily T2-weighted static-fluid sequences to evaluate the collecting systems and ureters, along with additional anatomical sequences. Contrast-enhanced excretory MRU was performed when clinically appropriate and not contraindicated. MRU findings were evaluated for urinary tract dilatation, masses, urothelial lesions, inflammatory or infective changes, obstructive pathology, and other relevant abnormalities.

Image interpretation and diagnostic comparison

CTU and MRU examinations were reviewed independently by experienced radiologists using a structured assessment format. The findings of each modality were documented separately. The reader assessing one modality was blinded to the findings of the other modality at the time of initial interpretation. Lesion-wise findings were recorded for each modality, including the presence or absence of urinary tract pathology and the suspected etiological category.

Because histopathological confirmation was not available for all etiologies, the final diagnostic reference standard was a composite reference standard consisting of CTU findings, MRU findings, clinical assessment, laboratory findings, operative or histopathological findings where available, and follow-up information when available. CTU was used as the principal imaging comparator for assessing the diagnostic performance of MRU; however, CTU also contributed to the composite reference standard. Therefore, incorporation bias could not be completely excluded, and the calculated CTU diagnostic performance estimates were interpreted descriptively within this cohort rather than as an independent validation of CTU accuracy.

The main outcome measures included lesion detection, etiological classification of hematuria, diagnostic accuracy parameters of CTU and MRU, and concordance between the two imaging modalities. Imaging findings were categorized as positive or negative for urinary tract pathology, and the detected etiologies were classified into clinically relevant groups, including urolithiasis, renal mass, urothelial carcinoma, infective or inflammatory pathology, and normal diagnostic assessment. This classification was used because these categories represent the major clinically actionable causes of hematuria and reflect the practical diagnostic pathway followed in routine urological and radiological evaluation.

Urolithiasis was classified when calculus disease was identified as the likely cause of hematuria. Renal mass included solid or complex renal lesions suspicious for neoplastic pathology. Urothelial carcinoma included suspected or confirmed lesions arising from the renal pelvis, ureter, or urinary bladder. Infective or inflammatory pathology included imaging and clinical findings consistent with urinary tract infection, pyelonephritis, cystitis, or related inflammatory disease. Patients without demonstrable urinary tract pathology on the final diagnostic assessment were classified as having a normal diagnostic assessment [1,2,4].

Statistical analysis

Data were entered into Microsoft Excel and analyzed using SPSS Statistics version 24 (IBM Corp., Armonk, NY). Continuous variables were summarized as means and standard deviations (SDs), while categorical variables were summarized as frequencies and percentages. Diagnostic performance was evaluated using true-positive, false-positive, true-negative, and false-negative values, along with sensitivity, specificity, positive predictive value, negative predictive value, and overall diagnostic accuracy. Exact 95% confidence intervals were calculated for diagnostic accuracy estimates where applicable. Concordance between CTU and MRU was assessed using observed agreement and Cohen’s kappa coefficient. A p-value of less than 0.05 was considered statistically significant where applicable.

Ethical considerations

The study was reviewed and approved by the Institutional Ethics Committee, Raipur Institute of Medical Sciences, Raipur, Chhattisgarh, India, with approval number IEC/RIMS/2024/0, dated August 14, 2024. Written informed consent was obtained from all participants before their inclusion in the study. Patient confidentiality was maintained throughout the study, and only de-identified data were used for analysis.

## Results

Demographic characteristics

A total of 35 patients with hematuria were included in the analysis. The age of the study population ranged from 27 to 63 years, with a mean age of 45.09 ± 10.91 years. The largest age group was 41-50 years, comprising 10 patients (28.6%), followed by the 31-40-year and 51-60-year age groups, with nine patients (25.7%) in each group. There were 21 males (60.0%) and 14 females (40.0%). Gross hematuria was present in 20 patients (57.1%), while microscopic hematuria was present in 15 patients (42.9%). A high-risk clinical profile was recorded in 19 patients (54.3%), while 16 patients (45.7%) were classified as low risk. The demographic and clinical characteristics are summarized in Table 2.

**Table 2:** Demographic and clinical characteristics of the study population Age distribution, sex distribution, clinical type of hematuria, and risk-profile distribution of patients included in the study

Variable	Frequency/value	Percentage
Total patients	35	100.0
Age range, years	27–63	Not applicable
Mean age, years	45.09 ± 10.91	Not applicable
Age group: 21–30 years	4	11.4
Age group: 31–40 years	9	25.7
Age group: 41–50 years	10	28.6
Age group: 51–60 years	9	25.7
Age group: 61–70 years	3	8.6
Male	21	60.0
Female	14	40.0
Gross hematuria	20	57.1
Microscopic hematuria	15	42.9
High-risk clinical profile	19	54.3
Low-risk clinical profile	16	45.7

Etiological spectrum of hematuria

A definite pathological etiology was identified in 30 of 35 patients, while five patients had no demonstrable abnormality on final diagnostic assessment. Infection was the most frequent etiology, observed in nine patients (25.7%), followed by renal mass in eight patients (22.9%), urothelial carcinoma in seven patients (20.0%), and urolithiasis in six patients (17.1%). Normal imaging findings were observed in five patients (14.3%). The etiological distribution of hematuria is shown in Table 3.

**Table 3:** Etiological distribution of hematuria Final etiological diagnosis based on imaging findings, clinical correlation, laboratory data, and available follow-up information

Etiology	Frequency	Percentage
Infection	9	25.7
Renal mass	8	22.9
Urothelial carcinoma	7	20.0
Urolithiasis	6	17.1
Normal diagnostic assessment	5	14.3
Total	35	100.0

Diagnostic accuracy analysis

Diagnostic performance was assessed using the final diagnostic categorization based on the composite reference standard. CTU identified all 30 patients with urinary tract pathology and correctly classified all five patients without demonstrable pathology in this cohort. CTU showed sensitivity of 100.0% (95% CI: 88.6-100.0), specificity of 100.0% (95% CI: 56.6-100.0), positive predictive value of 100.0%, negative predictive value of 100.0%, and overall accuracy of 100.0%. These point estimates should be interpreted in the context of the small sample size and wide confidence intervals, particularly for specificity.

MRU identified 26 of 30 patients with urinary tract pathology and correctly classified all five patients without demonstrable pathology. Four pathological cases detected on CTU were not detected on MRU. MRU showed sensitivity of 86.7% (95% CI: 70.3-94.7), specificity of 100.0% (95% CI: 56.6-100.0), positive predictive value of 100.0% (95% CI: 87.1-100.0), negative predictive value of 55.6% (95% CI: 26.7-81.1), and overall diagnostic accuracy of 88.6% (95% CI: 74.0-95.5). The diagnostic accuracy parameters of CTU and MRU are summarized in Table 4.

**Table 4:** Diagnostic performance of CTU and MRU Diagnostic accuracy parameters of computed tomography urography and magnetic resonance urography for detecting urinary tract pathology among patients with hematuria. True-positive, false-positive, true-negative, and false-negative values are expressed as frequency/total cohort with percentage. Sensitivity, specificity, predictive values, and overall diagnostic accuracy are expressed as percentages with 95% CIs where applicable CTU: computed tomography urography; MRU: magnetic resonance urography; CI: confidence interval

Parameter	CTU	MRU
True positive	30/35 (85.7%)	26/35 (74.3%)
False positive	0/35 (0.0%)	0/35 (0.0%)
True negative	5/35 (14.3%)	5/35 (14.3%)
False negative	0/35 (0.0%)	4/35 (11.4%)
Sensitivity	100.0% (95% CI: 88.6-100.0)	86.7% (95% CI: 70.3-94.7)
Specificity	100.0% (95% CI: 56.6-100.0)	100.0% (95% CI: 56.6-100.0)
Positive predictive value	100.0%	100.0% (95% CI: 87.1-100.0)
Negative predictive value	100.0%	55.6% (95% CI: 26.7-81.1)
Overall diagnostic accuracy	100.0%	88.6% (95% CI: 74.0-95.5)

Concordance between CTU and MRU

Agreement analysis demonstrated concordant CTU and MRU findings in 31 of 35 patients, corresponding to an observed agreement of 88.6%. Both CTU and MRU were positive in 26 patients, and both were negative in five patients. Discordance was observed in four patients, all of whom were positive on CTU but negative on MRU. No patient was negative on CTU and positive on MRU. Cohen’s kappa coefficient was 0.65, indicating substantial agreement beyond chance, and the agreement was statistically significant (z = 4.11, p < 0.001). The concordance matrix is shown in Table 5.

**Table 5:** Concordance between CTU and MRU Agreement between CTU and MRU for detection of urinary tract pathology. Values are expressed as frequency/total cohort with percentage. Observed agreement was 88.6%, and Cohen’s kappa coefficient was 0.65, indicating substantial agreement beyond chance CTU: computed tomography urography; MRU: magnetic resonance urography

CTU finding	MRU negative	MRU positive	Total
CTU negative	5/35 (14.3%)	0/35 (0.0%)	5/35 (14.3%)
CTU positive	4/35 (11.4%)	26/35 (74.3%)	30/35 (85.7%)
Total	9/35 (25.7%)	26/35 (74.3%)	35/35 (100.0%)

Interpretation of diagnostic findings

The findings indicate that CTU had the highest sensitivity and negative predictive value in the present cohort, supporting its role as the more comprehensive modality for excluding urinary tract pathology in patients with hematuria. MRU demonstrated excellent specificity and positive predictive value, indicating that positive MRU findings were highly reliable. However, the lower sensitivity and negative predictive value of MRU indicate that a negative MRU result should be interpreted cautiously, particularly when clinical suspicion remains high. The confidence intervals, especially for specificity, are wide because only five patients had no demonstrable pathology, and this limitation should be considered while interpreting the results.

## Discussion

Principal findings

The present study compared CTU and MRU in patients presenting with hematuria and found that CTU demonstrated the highest diagnostic performance across the evaluated etiological spectrum. CTU detected all pathological cases in the study cohort, whereas MRU showed high specificity and positive predictive value but comparatively lower sensitivity and negative predictive value. The agreement between CTU and MRU was substantial, indicating that MRU can provide clinically useful information in selected patients, although it should not be considered a complete substitute for CTU in all cases.

The etiological spectrum in this study reflected the heterogeneous nature of hematuria. Infection was the most common cause, followed by renal mass, urothelial carcinoma, and urolithiasis. This distribution reinforces the clinical importance of imaging in hematuria evaluation, as the underlying cause may range from benign inflammatory disease to clinically significant malignancy [1,2]. The presence of a sizable proportion of renal masses and urothelial carcinomas in this cohort also emphasizes that hematuria should not be treated as a trivial symptom, particularly when it is persistent, recurrent, painless, or unexplained [1,5].

CTU performance and clinical relevance

CTU demonstrated excellent diagnostic performance in the present study, with complete detection of the 30 pathological cases and correct classification of the five negative cases. This finding is consistent with the established role of CTU as a comprehensive imaging modality for hematuria evaluation [3,5,6]. Its strength lies in its ability to evaluate the renal parenchyma, collecting systems, ureters, and urinary bladder in a single multiphasic examination. The non-contrast phase is useful for detecting urinary calculi, while nephrographic and excretory phase imaging improves the assessment of renal masses, urothelial lesions, filling defects, and obstructive pathology [3,7,8].

The strong performance of CTU in the present cohort is clinically important because missed pathology in hematuria evaluation may delay the diagnosis of malignancy or obstructive disease [1,2,5]. CTU was especially useful for detecting urothelial lesions and urolithiasis, which are areas where high spatial resolution and contrast-opacified excretory imaging are advantageous [7-9]. Previous studies have specifically reported the value of CTU for urinary tract abnormalities, upper tract urothelial neoplasms, bladder cancer, transitional cell carcinoma, and urinary tract neoplasms in patients with hematuria [9-13].

However, the apparent perfect diagnostic performance of CTU in this study must be interpreted cautiously. The sample size was limited, and only five patients had no demonstrable pathology. As a result, the confidence interval for specificity was wide despite a point estimate of 100%. Therefore, the results support the strong diagnostic value of CTU but should not be interpreted as evidence that CTU is infallible. In clinical practice, CTU accuracy remains dependent on appropriate patient preparation, adequate urinary tract opacification, acquisition protocol, lesion size, reader experience, and availability of confirmatory clinical or pathological correlation [3,6-8].

MRU performance and diagnostic role

MRU showed high specificity and positive predictive value in this study, indicating that positive MRU findings were reliable. MRU identified all renal masses and infective etiologies in the cohort, supporting its value in evaluating soft-tissue and parenchymal abnormalities. The superior soft-tissue contrast of MRU is a recognized advantage, particularly in renal mass characterization, periureteric disease, inflammatory processes, urinary tract obstruction, and patients in whom iodinated contrast or radiation exposure is undesirable [14-16].

Despite these advantages, MRU had lower sensitivity than CTU in the present study. Four pathological cases detected by CTU were not detected by MRU, and the missed cases were mainly related to urothelial pathology. This finding is consistent with previous literature indicating that MRU may be less sensitive for subtle urothelial lesions when compared with CTU, while remaining useful for selected upper urinary tract and soft-tissue assessment [14,15,18,19]. The relatively low negative predictive value of MRU in this study is particularly important. It indicates that a negative MRU examination should not be used alone to exclude urinary tract pathology when clinical suspicion remains high [6,14,18,19].

The practical interpretation is that MRU should be viewed as a complementary or alternative modality rather than a universal replacement for CTU [6,14,19,20]. It may be especially useful in selected patients requiring radiation avoidance, those requiring repeated imaging, and those in whom iodinated contrast administration is undesirable [5,6,14,19]. In contrast, CTU remains preferable in high-risk patients, especially when urothelial carcinoma, ureteric pathology, or urolithiasis is strongly suspected [3,7-13].

Concordance between CTU and MRU

The observed agreement between CTU and MRU was 88.6%, and Cohen’s kappa coefficient indicated substantial agreement beyond chance. This suggests that MRU findings corresponded well with CTU in most patients. However, the direction of discordance is clinically meaningful. All discordant cases were positive on CTU and negative on MRU, while there were no cases that were negative on CTU and positive on MRU. This pattern suggests that MRU was unlikely to overcall disease in this cohort but was more likely to miss selected lesions, particularly urothelial abnormalities.

The concordance results therefore support a selective role for MRU. When MRU demonstrates a definite abnormality, particularly a renal mass, infection-related abnormality, or obstructive process, the finding is likely to be clinically relevant [14-20]. However, when MRU is negative in a patient with persistent hematuria or high-risk clinical features, CTU or cystoscopic evaluation should still be considered depending on the suspected site of disease and overall clinical context [1,5,9-13].

Comparison with previous studies

The findings of the present study are broadly consistent with earlier comparative work on CTU and MRU. Previous studies have shown that CTU provides high-resolution multiphasic assessment of the urinary tract and is useful for detecting calculi, renal masses, urothelial lesions, and obstructive pathology in patients with hematuria [3,6-8]. Studies focused on urothelial malignancy have also shown the diagnostic value of CTU for upper tract urothelial neoplasms, bladder cancer, transitional cell carcinoma, and urinary tract neoplasms [9-13].

MRU studies have shown that heavily T2-weighted static-fluid sequences and contrast-enhanced excretory techniques can provide useful anatomical and functional information regarding the collecting systems and ureters [14-17]. Comparative and protocol-based studies have also reported that MRU can provide useful diagnostic information in selected urinary tract conditions, particularly when radiation avoidance or iodinated contrast avoidance is clinically important [16-20].

The present study adds practical data from a tertiary care setting in which both modalities were assessed in the same patient cohort. This head-to-head design reduces between-patient variability and allows more direct assessment of modality-specific performance. The results support the continued role of CTU as the primary comprehensive imaging test in many patients with hematuria, while also reinforcing the potential value of MRU in carefully selected patients for whom radiation avoidance or iodinated contrast avoidance is clinically important [5,6,16-20].

Clinical implications

The study findings support a patient-specific imaging strategy rather than a uniform imaging approach for all patients with hematuria [1,4,5]. CTU may remain the preferred modality when comprehensive evaluation is required, particularly in patients at higher risk for malignancy, patients with suspected urothelial disease, and patients with possible urolithiasis [3,5,7-13]. MRU may be considered when the expected diagnostic target is renal parenchymal or soft-tissue pathology, when CTU is contraindicated, or when radiation exposure is a major concern [6,14-20].

Adult hematuria has variable clinical significance, and structured evaluation remains important because underlying causes may range from benign disease to clinically significant urological pathology [21-24]. Recent CTU literature also emphasizes that imaging protocols may be adapted according to patient age, clinical context, contrast exposure, and the potential relevance of incidental findings [25,26]. Additional imaging reviews and AUA best-practice recommendations support appropriate cross-sectional imaging, cystoscopic evaluation, and follow-up in selected patients with hematuria [27,28]. Further evidence has questioned whether CT urography is required for all patients presenting with hematuria, supporting a more selective and patient-specific imaging approach [29]. Radiation exposure from CT remains an important safety consideration when selecting imaging strategies, particularly in younger patients or those requiring repeated examinations [30].

This approach is clinically relevant because hematuria evaluation often requires balancing diagnostic yield against patient safety [1,4,5]. CTU offers speed, availability, and high diagnostic confidence, but at the cost of radiation and iodinated contrast exposure [3,5,6,30]. MRU avoids ionizing radiation and provides strong soft-tissue contrast, but its longer acquisition time, susceptibility to motion artifacts, limited availability, and lower sensitivity for selected lesions restrict its use as a first-line test in all patients [6,14-20]. A tailored algorithm using clinical risk factors, renal function, patient age, pregnancy status, contrast allergy, and suspected pathology may provide the most rational approach [1,4,5,25,26,29,30].

Strengths of the study

The main strength of this study is that both CTU and MRU were performed in the same patient cohort, allowing direct comparison of diagnostic performance. The use of consecutive sampling reduced selection bias among eligible patients undergoing both examinations. The study also assessed not only descriptive imaging findings but also diagnostic accuracy parameters and agreement statistics, making the results more clinically interpretable. In addition, the study included a varied etiological spectrum of hematuria, including infection, renal mass, urothelial carcinoma, urolithiasis, and normal imaging findings.

Limitations

This study has several limitations. First, it was conducted at a single tertiary care institution with a relatively small cohort, which limits the precision and generalizability of the diagnostic performance estimates. Only five patients were classified as having no demonstrable urinary tract pathology; therefore, estimates such as 100% specificity and positive predictive value are based on a small number of negative cases and should be interpreted with caution despite their high point estimates. Second, histopathological or other independent confirmatory evidence was not available for all etiologies, and a composite reference standard was therefore used. Because CTU contributed to this composite reference standard while its diagnostic performance was also calculated against that standard, incorporation bias could not be excluded, and the apparent 100% CTU performance should not be interpreted as an independent validation of CTU accuracy.

Third, the principal diagnostic analysis combined heterogeneous etiologies, including urolithiasis, renal masses, urothelial carcinoma, and infective or inflammatory pathology, into a binary pathological-versus-normal endpoint. This may obscure important modality-specific differences, particularly because the relative strengths and limitations of CTU and MRU differ across these disease categories. Fourth, eligibility required patients to complete both CTU and MRU; consequently, patients unable to undergo either examination because of renal dysfunction, contrast-related risks, MRI contraindications, or other clinical factors were excluded, which may limit applicability to some populations in whom alternative imaging is clinically relevant. MRU performance may vary according to scanner strength, acquisition protocol, patient cooperation, radiologist experience, and sequence optimization [14-20]. Finally, long-term follow-up was not available for all patients, which may have limited independent confirmation of initially negative examinations.

Overall interpretation

Overall, the findings indicate that CTU remains the most comprehensive imaging modality for hematuria evaluation in this cohort, particularly for excluding pathology and detecting urothelial lesions [3,5,7-13]. MRU demonstrated high specificity and substantial agreement with CTU, supporting its use as a radiation-free alternative in selected patients [14-20]. However, the lower sensitivity and negative predictive value of MRU indicate that negative MRU findings should be interpreted cautiously in patients with persistent symptoms or high-risk clinical features [1,5,14,18,19]. Larger multicentric studies with standardized MRU protocols, histopathological correlation, and long-term follow-up are required to better define the role of MRU in hematuria imaging algorithms.

## Conclusions

In this prospective paired cohort, MRU demonstrated substantial agreement with CTU for the detection of urinary tract pathology in patients with hematuria but failed to detect four abnormalities identified on CTU. MRU showed high observed specificity and positive predictive value, although these estimates should be interpreted cautiously because of the small number of reference-negative patients. CTU showed higher observed sensitivity and negative predictive value within this cohort; however, its apparent 100% diagnostic performance should not be regarded as an independent estimate of CTU accuracy because CTU contributed to the composite reference standard. These findings suggest that MRU may have a useful alternative or complementary role in selected patients, particularly when avoidance of ionizing radiation or iodinated contrast is clinically important. However, a negative MRU examination should be interpreted cautiously when clinical suspicion for urinary tract pathology remains high. Larger studies using independent reference standards, standardized imaging protocols, and lesion-specific analysis are needed to establish the comparative diagnostic performance of CTU and MRU more definitively.
